# Does local vancomycin powder impregnated with autogenous bone graft and bone substitute decrease the risk of deep surgical site infection in degenerative lumbar spine fusion surgery?—An ambispective study

**DOI:** 10.1186/s12891-022-05802-y

**Published:** 2022-09-10

**Authors:** Po-Hsin Chou, Hsi-Hsien Lin, Yu-Cheng Yao, Ming-Chau Chang, Chien-Lin Liu, Shih-Tien Wang

**Affiliations:** 1 School of Medicine, National Yang Ming Chiao Tung University, Taipei, Taiwan; 2 Department of Orthopedics and Traumatology, Taipei Veterans General Hospital, Taipei, Taiwan

**Keywords:** Deep surgical site infection, Vancomycin, Local delivery system, Degenerative lumbar fusion surgery

## Abstract

**Background:**

Deep surgical site infection (DSSI) is one of the most challenging complications in lumbar fusion surgery. Few investigations examined the effect of vancomycin powder mixed with autogenic bone graft (ABG) and bone substitutes on preventing DSSI in degenerative lumbar fusion surgeries as well as any interference with bony fusion. The aim of the study was to investigate the effects of ABG along with bone substitutes as a local vancomycin delivery system on preventing DSSI in lumbar instrumented fusion and compared with those who did not use vancomycin powder.

**Methods:**

From January, 2015 through December, 2015, a one-year prospective study using vancomycin powder mixed with ABG and bone substitute for degenerative lumbar fusion surgeries as vancomycin (V) group, 1 gm vancomycin for 2 and 3-level, and 2 gm for more than 3-level instrumentation. From December, 2013 through December 2014, patients received degenerative lumbar fusion surgeries without using vancomycin before the vancomycin protocol were retrospectively enrolled as non-vancomycin (NV) group. Vancomycin concentration was checked at post-operative days 1 and 3 for both the serum and drainage. Patients’ demographic data, microbiology reports, fusion status and functional outcomes were evaluated.

**Results:**

One hundred and ten patients were enrolled prospectively in the V group, and 86 for the NV group. After an average 41 months follow-up (range, 36–54), 3 patients (3.48%) developed postoperative DSSIs in the NV group, thereby requiring revision surgeries and parenteral antibiotics treatment versus no DSSIs (0%, 0/100) in the V group. (*p* = 0.048). The postoperative serum vancomycin levels were undetectable and no vancomycin related side effects was encountered. The mean vancomycin concentration of drainage at postoperative days 1 and 3 were 517.96 ± 174.4 and 220.14 ± 102.3 μg/mL, respectively. At final follow-up, there was no statistical difference observed in terms of clinical and radiologic outcomes.

**Conclusions:**

Our vancomycin protocol may reduce the incidence of DSSI in degenerative lumbar fusion surgery without affecting bony fusion.

**Level of Evidence:**

Level III ambispective comparative study.

## Background

Deep Surgical site infection (DSSI) is one of the most serious problems in orthopedic surgery and can be more complicated with implants in the joints or bones. To reduce the incidence of DSSI, delivery of local antibiotics has become popular in orthopedic surgery [1]. The benefit of local antibiotic delivery is obtaining high levels of antibiotics without increasing systemic toxicity [1]. Bone cement is one of the gold materials for local antibiotic delivery in orthopedic surgery [1]. Moreover, another delivery system, such as bone graft, either autograft, allograft or synthetic bone, has been clinically used in treatment of infective non-union of tibia [2, 3].

The infection rates following spinal instrumented fusion have been reported up to 7.7% [4, 5]. In order to reduce DSSI following spinal instrumented fusion surgery, local application of vancomycin powder on superficial or subfascial tissue or both [6–10] has been reported with successful results. However, Eder C [11] reported osteoblast proliferation was significantly inhibited with a vancomycin level above 3 mg/cm^2^ and cell death exceeding 6 mg/cm^2^ in a in vitro study. Besides, significant disability and pain have been reported in patients with pseudarthrosis following spinal instrumented fusion surgery [12]. Therefore, it is important for spine surgeons to find a balance between decreasing DSSI and avoiding non-union in terms of local application of vancomycin.

Moreover, papers regarding the effect of autogenic bone graft (ABG) as a local vancomycin delivery system to prevent DSSI in degenerative lumbar spinal fusion surgery was not much [8, 13]. Accordingly, it is important for spine surgeons to investigate the effects of vancomycin power mixing with ABG not only on DSSI prevention but bony fusion interference, especially when applying vancomycin powder in the degenerative lumbar fusion surgery. Therefore, we designed an ambispective study to examine the effects of autogenic bone graft along with bone substitute as a local vancomycin delivery system on preventing DSSI in instrumented fusion for degenerative lumbar spinal disorders, functional outcomes and incidence of non-union were also investigated and compared with those who did not use vancomycin powder.

## Methods

**Fig. 1:**
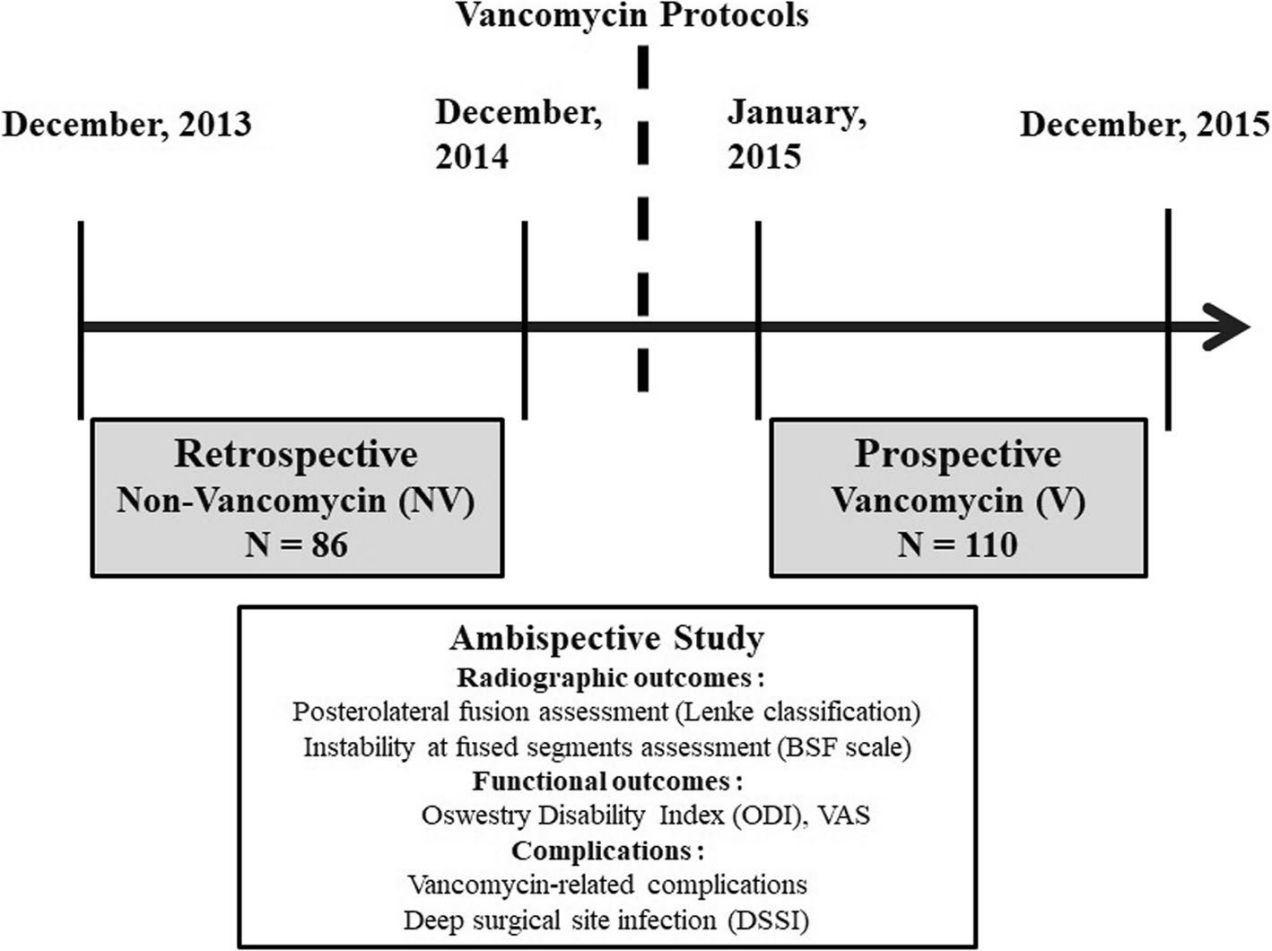
The study design of this ambispective study. The study was composed of retrospective study (non-vancomycin, *N* = 86) before the vancomycin protocol and prospective study (vancomycin, *N* = 110) after the protocol set up

From January through December, 2015, a one-year prospective study was conducted using vancomycin powder (VP) mixed with autogenous bone graft and bone substitute for those patients with degenerative lumbar disorders who needed surgical intervention with posterior decompression, instrumentation and fusion and were grouped as V group after getting the approval of internal review board at our hospital. Then a retrospective study was conducted with the patients not using VP and grouped as NV group from December, 2013 through December, 2014 (Fig. 1). The indications of surgery were persistent back and radicular pain with neurologic claudication and failure of conservative treatment for at least 3 months. All instrumentation including transpedicle screws and transforaminal lumbar interbody fusion (TLIF) cage was approved by the National Health Insurance Bureau and done by one senior surgeon (S-T W). The patients who had previous spinal surgery or history of allergic reaction to vancomycin were excluded. Patients’ demographic data, microbiology reports, fusion status and functional outcomes regarding Oswestry disability index (ODI) and visual analogue scale (VAS) for back and leg pain were recorded and analyzed preoperatively and at latest follow-up.

Prophylactic antibiotic was given intravenously with 1 gm cephalosporin 30 min before skin incision and redosing at a 4-h interval intra-operatively. After operation, 1 gm cephalosporin was given at an 8-h interval and gentamicin 80 mg at a 12-h interval for three days. Traditional open posterior decompression, instrumentation and fusion were carried out with autogenous bone graft (ABG) from the bone chips of decompressed laminae and spinous processes and mixed with β-tricalcium phosphate bone substitute (chronOS ®, DePuy Synthes, West Chester, PA, USA) at a 1:1 volume ratio. Then, 1 gm vancomycin powder (Gentle Pharmaceutical Co., Yunlin, Taiwan) was mixed homogenously with the mixture of ABG and bone substitute for 2 or 3-level and 2 gm for more than 3-level. In order to prevent vancomycin being washed out by blood, the mixture was left undisturbed for at least 30 min to allow the vancomycin powder being adhered adequately to the mixture of bone graft.

**Fig. 2:**
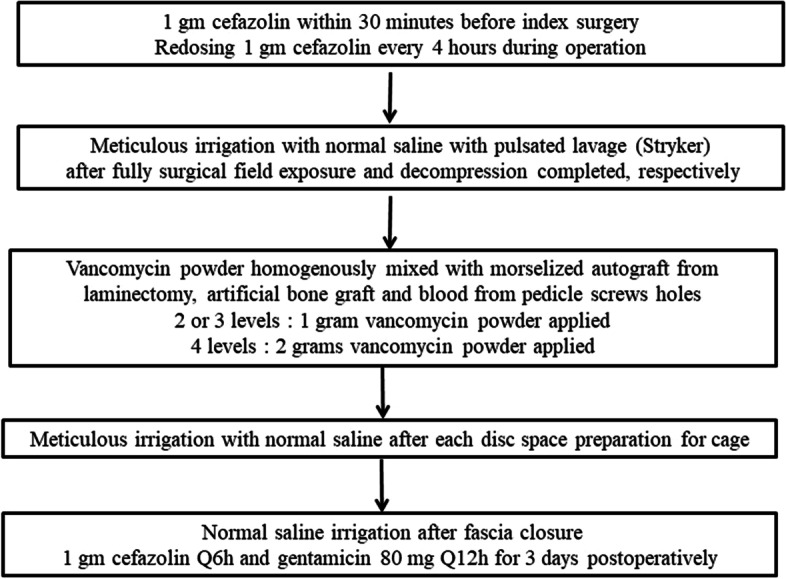
The vancomycin protocol and our methods of infection were controlled step by step during the whole operation

Intraoperative meticulous irrigation with normal saline using a pulsatile lavage system (Interpulse; Stryker Corp, Kalamazoo, MI, USA) was routinely performed for both V and NV groups throughout the whole procedure (Fig. 2). Finally, the wound was closed in the usual manner with a suction drainage tube left in. Patients were allowed ambulating with an orthosis at post-op days 3 or 4 after removal of drainage.

Vancomycin concentrations in the V group were checked at post-operative day 1 (POD1) and 3 (POD3) following surgery for both serum and surgical site, which was collected from the drainage, and were analyzed by Architect iVancomycin (Abbott, Wiesbaden, Germany) using the Architect i1000 SR analyzer (Abbott Laboratories, North Chicago, IL, USA). Architect iVancomycin is an in vitro chemiluminescent microparticle immunoassay for the quantitative measurement of vancomycin in human serum or plasma.

After the operation, all patients were followed up at post-operative 3- month, 6–month, 12-month and annually. Dynamic flexion and extension lateral radiographs were performed at post-operative 2-year to evaluate whether solid fusion was achieved. Radiographic pedicle screw loosening was defined as a 1 mm or greater radiolucent halo surrounding the pedicle screw (halo sign and double halo sign), which was adopted from Sanden B et al. [14].

The posterolateral fusion was evaluated using Lenke criteria [15] (Table 1). The definition of cage fusion was bridging bone across the disc space from one vertebrae to the adjacent level using Brantigan, Steffee, Fraser (BSF) scale [16]. Two spine surgeons (P–H Chou and Y-C Yao), who were not involved in the surgery, evaluated the fusion status, respectively. Follow-up CT scan was not routinely arranged for fusion evaluation because of cost reduction, reduction of radiation exposure, artifacts by the metallic implants and the policy of Taiwan’s National Health Insurance.

**Table 1:** Lenke classification for lumbar posterolateral fusion assessments

Grading	Fusion	Description
A	Solid	big trabeculated fusion, bilaterally
B	Possibly Solid	big fusion mass at unilateral with a small fusion mass at the contralateral side
C	Probably Not Solid	small, thin fusion masses bilaterally with apparent crack
D	Definitely Not Solid	graft resorption bilaterally or fusion mass with an obvious bilateral pseudarthrosis

The fusion criteria was adopted from J Spinal Disord 1992;5:433–42

Once DSSI was suspected, magnetic resonance image (MRI) of lumbar spine and serum C-reactive protein (CRP) were checked. The definition of DSSI was defined as "pedicle screw fluid sign" according to Kimura H et al. [17] and TLIF cage as well. CT-guided biopsy was arranged and the diagnosis was confirmed by either histopathology or bacterial culture. All samples from CT-guided biopsy were placed on 10% aerobic and anaerobic blood agar plates. For identification of Staphylococcus aureus, matrix-assisted laser desorption-ionization time-of-flight mass spectrometry (bioMérieux) was used. Susceptibilities of Staphylococcus aureus isolated to antimicrobial agents were determined by Vitek2 system (bioMérieux). The antimicrobial susceptibilities were interpreted according to the Clinical and Laboratory Standards Institutes (CLSI) breakpoint [18]. Specimen from CT-guided biopsy were fixed in 10% neutral buffered formalin and decalcified by immersion in Shandon TBD-1 rapid decalcifier containing 10% hydrochloric acid (Thermo Electron Corporation) for 1 h. The decalcified material was then processed and embedded in paraffin, sectioned in 3 to 5 μm slices and stained with hematoxylin and eosin. Stained sections were examined with attention by experienced pathologists using light microscopy under lower-powered (X40) and high-powered (X400), respectively.

Once the diagnosis of DSSI being established, effective intravenous antibiotics were administrated for at least 6 weeks or until the ESR and CRP level returned to normal, which were checked weekly, and were followed by oral antibiotics for another 6 weeks.

Statistical analysis was performed using SPSS for windows (version 15.0; SPSS, Chicago, Illinois, 1999). Student’s t test was used for numerical data and chi square test for categorical data. A p value less than 0.05 considered statistical significance. To determine whether these tests were appropriately powered, power analysis was also performed using G*Power software (Heinrich-Heine Universität Düsseldorf, Düsseldorf, Germany).

## Results

From January through December, 2015, 110 patients were prospectively enrolled as vancomycin (V) group, and 86 patients without using intra-operative vancomycin (non-vancomycin, NV group) were retrospectively enrolled from December, 2013 through December, 2014. The overall average age of the patients was 73.1 year-old (range, 49 to 82) at operation, 73.7 year-old for the V group and 72.5 year-old for the NV group. There was no statistical significance between these two groups regarding pre-operative demographic data and functional outcomes. The mean follow-up time was 38 months and 53 months for the V and NV groups, respectively, which was significantly longer for the NV group. (Table 2).

**Table 2:** Pre-operative demographic data between two groups

	**Vancomycin (V)**	**Non-Vancomycin (NV)**	***P* value**
**No. of Patients**	110	86	
**Mean Age at Op. (years)**	73.7 ± 9.8 (49–82)	72.5 ± 10.6 (55–81)	0.413
**Body Mass Index (BMI)**	21.8 ± 3.5 (15.9–32.9)	22.2 ± 3.8 (16.7–34.6)	0.445
**Gender**			0.689
Male	48	40	
Female	62	46	
**Pathologic Lesions**			0.927
2 levels	36	27	
3 levels	58	47	
4 levels	16	12	
**Co-morbidities**			0.577
Diabetes mellitus	23	14	
BMI > 30	2	3	
Rheumatoid arthritis	0	1	
Steroid Used	5	4	
Smoker	26	14	
**Functional Outcomes**			
Visual analogue scale over back	4.2 ± 2.1(2–8)	4.3 ± 2.4 (1–8)	0.746
Visual analogue scale over leg	5.0 ± 1.6 (4–7)	4.9 ± 1.8 (4–8)	0.682
Oswestry Disability Index (ODI)	54.8 ± 12.6 (38–70)	55.7 ± 13.3(24–74)	0.629
**Mean follow-up times (months)**	38.3 ± 6.2 (36–48)	53.1 ± 5.7(48–60)	**0.000**

All numbers were presented with mean ± standard deviation (range), with the range in parentheses

The average vancomycin concentrations obtained from the drain were 517.96 ± 161.72 μg/mL (range, 107.9–932.4) and 220.14 ± 102.3 μg/mL (range, 74.3–591.2) at post-operative day 1 and 3 (POD 1 and POD 3), respectively, whereas vancomycin was undetectable in the serum (Table 3). There was no adverse event related to the local application of vancomycin such as red man syndrome, allergic reaction, ototoxicity or renal toxicity.

**Table 3:** Vancomycin levels in serum and drain in vancomycin group

**Post-operative day**		**1st (POD 1)**	**3rd (POD 3)**
Vancomycin	**Drain**	517.96 ± 161.72 (107.9–932.4)	220.14 ± 102.3 (74.3–591.2)
concentration	**Serum**	< 0.24	< 0.24

*N* = 110 patients in vancomycin group, the unit of vancomycin concentration wasμg/mL

The results were presented as mean ± SD with the range in parentheses

We used ELISA method to measure the vancomycin concentration

No infection (0/110, 0%) was encountered in the V group, but 3 patients (3/86, 3.48%) had DSSI in the NV group, which was statistically significant higher being observed in the NV group (*P* = 0.048) (Table 4). All 3 patients had severe back pain after surgery and fluid accumulation sign around screws or TLIF cage in the MRI. Methicillin-resistant Staphylococcus aureus (MRSA) was cultured within three months of the index operation in two patients, and the other patient had a negative culture and was diagnosed by histopathology. One patient needed an anterior surgery to remove the loosening cage and fusion with tricortical iliac strut graft. Another one patient only needed removal of pedicle screws, and the other one could be treated with parenteral antibiotic alone without removal of implants.

**Table 4:** Surgical results and complications between two groups

	**Vancomycin (V)**	**No Vancomycin (NV)**	***P* value**
**Numbers of Patients**	110	86	
**Blood Loss (mL)**	282 ± 280 (150–1100)	297 ± 295 (140–1000)	0.717
**Operative Times (mins)**	281 ± 72 (200–400)	285 ± 63 (220–410)	0.687
**Deep Surgical Site Infection (DSSI)**	0 (0%)	3 (3.48%)	0.048*
**Surgical-Related Complications (patients)**			0.350
Screws breakage or loosening	2	4	
Cage Dislodge or migration	0	1	
Incidental Durotomy	2	2	
**Vancomycin-Related Complications (patients)**			
Red Man syndrome	0	NA	
Allergic reaction	0	NA	
Renal toxicity	0	NA	
Ototoxicity or transient hearing loss	0	NA	
Systemic Absorption (Detectable Serum Vancomycin)	0	NA	
**Functional Outcomes**			
Visual analogue scale (VAS) over back	1.8 ± 1.3 (1–5)	1.7 ± 1.2 (0–4)	0.581
Visual analogue scale (VAS) over leg	1.3 ± 0.4 (1–4)	1.2 ± 0.5 (0–3)	0.121
Oswestry Disability Index (ODI)	31.8 ± 9.6 (16–48)	32.9 ± 10.1 (24–50)	0.438

The percentage was presented in parentheses, NA meant Non-appreciable, f/u meant follow-up

2 patients had S1 screws loosening in the V group at latest f/u

2 patients had S1 screws loosening in the NV group at latest f/u. (2: S1 screws, 2: infective non-union)

The mean operative time was 281 and 285 min in the V and NV groups, respectively (*P* = 0.687). The mean estimated blood loss was 282 and 297 mL in the V and NV groups, respectively (*P* = 0.717) (Table 2). The surgical complications besides DSSI were similar between the two groups (*P* = 0.350) (Table 4): 2 patients experienced screws breakage or loosening in both groups, and 2 in the NV group due to DSSI.

Total 232 cages were placed in the 196 patients with mean 1.18 cages insertion (range, 0–3) in each operated patient. According to the BSF scale, the interbody fusion rates were similar between the two groups (*p* = 0.436). One patient was excluded in the NV group due to DSSI and underwent implant removal surgery. By the Lenke classification, the posterolateral fusion rates were also similar between the two groups (*P* = 0.563) (Table 5). The functional outcomes regarding ODI were similar between these two groups at latest follow-up (*P* = 0.463) (Table 4). The visual analogue scale (VAS) for back and leg pain were also similar between two groups (*P* = 0.581 and *P* = 0.121), respectively.

**Table 5:** Results of bone fusion at latest follow-up between two groups

	**Vancomycin (V)**	**No Vancomycin (NV)**	***P*** **value**
**Numbers of Patients**	110	86	
**Numbers of Discs with Cages Insertion**	132	100	
**Posterolateral Fusion Evaluated by Lenke Classification (of patients)**			0.563
A (Definite Solid)	40	31	
B (Possibly Solid)	29	24	
C (Probably Not Solid)	38	27	
D (Definitely Not Solid)	3	4	
**Interbody fusion evaluated by Brantigan, Steffee and Fraser definition (of cages)**			0.463
BSF-1 (radiographical pseudarthrosis)	0	1	
BSF-2 (radiographical locked pseudarthrosis)	11	10	
BSF-3 (radiographical fusion)	121	89	

+ 1 patient underwent cage removal surgery due to infective non-union and loosening during follow-up

## Discussion

Postoperative DSSI following spinal fusion surgery is a challenging complication with a potentially catastrophic outcome, as well as significantly increases burden to the patient, patient’s family, and the health-care system. The most common organism isolated from DSSI following spinal fusion surgery is Staphylococcus aureus [19]. However, parenteral vancomycin usage was not as effective as cephalosporin in preventing SSIs in clean orthopedic surgery [20]. Besides, sides effects, such as infusion-related toxicities, nephrotoxicity, red man syndrome and ototoxicity, following parenteral vancomycin could be commonly occurred, even within therapeutic concentration [21]. Although vancomycin impregnated cement is one of the convincing methods to prevent deep infection during knee arthroplasty [22], the role of intra-wound vancomycin power (VP) on DSSI prophylaxis in degenerative lumbar spine fusion surgery is still elusive [6–10, 23].

Bone grafting as a deliver system for VP as adjuvant for DSSI prophylaxis has been reported [8, 13], which was similar to our protocol. Gans I [13] reported 500 mg VP was distributed subfascially and mixed with bone graft in pediatric spine deformity surgery regarding fusion, growing rod, and vertical expandable prosthetic titanium rib (VEPTR). However, the paper focused on the vancomycin-related systemic safety concerns such as anaphylaxis, nephrotoxicity, red man syndrome thrombophlebitis or rash for local application of VP in pediatric patients and did not report the impact of VP on bone fusion. Three (3.4%) in the totally 87 operated pediatric deformity correction still got DSSI at 1 to 2-month postoperatively in the cases series report.

Sweet FA et al. [8] reported a retrospective cohort study of applying 2 gm VP locally in almost all kinds of spine instrumented fusion surgeries including transforaminal lumbar interbody fusion, revision surgery, osteotomy, adolescent idiopathic scoliosis, adult scoliosis, trauma and tumor. They spread 1 gm VP deeply and superficially, and the other 1 gm VP was mixed with the bone grafting materials, which was different to our vancomycin protocol, including diversity of the diseases and sprinkling in the wound. In current study, we only focused on degenerative lumbar fusion surgery, and employed VP mixed with the bone grafting materials without sprinkling in the wound. Moreover, the definition of pseudarthrosis was not clearly described in the Sweet’s study [8], which was a major concern for spine surgeons when using VP locally. In our study, we adopted the fusion criteria using Lenke criteria [15] and BSF scale [16] for posterolateral and anterior interbody fusions respectively, which were widely accepted in the literature besides CT scan.

The overall incidences of DSSI in selective degenerative lumbar fusion surgery, adult spine deformity correction, spine trauma surgery and revision instrumented lumbar fusion ranged from 2.8% to 6% [24], 3.5% to 4.5% [25, 26], 3% to 9.4% [27, 28] and 2.2% to 4.5% [29, 30], respectively. The incidence of DSSI in selective degenerative lumbar fusion surgery is not an uncommon complication, which may have devastating consequence, and spine surgeons need to make an early diagnosis if any clinically suspicious.

Ghobrial GM [31] reported intra-wound vancomycin provided selective pressure with increased gram negative and polymicrobial infection. Chotai S reported [32] the occurrence of DSS caused by S aureus was lower in the V group as compared to those in the NV group (32% vs 65%). A gram-negative pathogen was detected in 28% and 12.5% of patients with DSSI in the V and NV groups, respectively. The incidence of polymicrobial (mixed with anaerobic and aerobic) was similar between two groups (5% for NV group, 4% for V group). Accordingly, significant difference of cultured organisms was observed in the vancomycin group [31, 32]. Neither these papers [31, 32] nor our series were investigated any vancomycin-resistant organisms in DSSI. Clinicians have to be aware of vancomycin-related selective pressure and immune-burden to avoid resistant organism and find a dynamic balance between DSSI prevention and local antibiotics application.

Minimal inhibitory concentration (MIC) is defined as the lowest concentration of antimicrobial that will inhibit the visible growth of microorganism following overnight incubation [33]. The mean MIC of vancomycin for MRSA has been reported 1.5–2 μg/mL [34]. In our study, the average vancomycin levels from the surgical site were 517.96 ± 161.72 and 220.14 ± 102.3 μg/mL at POD 1 and POD 3 respectively, which was much higher than the MIC of MRSA and might explain the effect of vancomycin on preventing DSSI postoperatively. Moreover, an undetectable serum vancomycin concentration may explain the little effects on systemic toxicity.

Regarding the inhibition of pre-osteoblast and osteoblast proliferation, three in vitro studies have been reported the vancomycin concentration greater the 3 mg/cm^2^, 2000 and 5000 μg/mL could inhibit proliferation of pre-osteoblast and osteoblast [11, 35, 36], which might lead to nonunion in vivo. The local vancomycin concentrations at POD 1 were 462 and 251 μg/mL have been reported by Sweet FA [8] and Armaghani [37], respectively, and 128 μg/mL at POD 3 by Sweet FA [8]. In our study, the average vancomycin levels were 517.96 ± 161.72 μg/mL (range, 107.9–932.4) and 220.14 ± 102.3 μg/mL (range, 74.3–591.23) at POD 1 and POD 3, respectively, which did not reach the inhibitory concentration for osteoblast. Therefore, the mixture of ABG and bone substitute could serve as a local vancomycin delivery system to maintain high vancomycin concentrations without jeopardizing bony fusion.

There are several drawbacks in our study including patients’ number is not enough to reach and adequate power and draw a solid conclusion. CT scan was not use for fusion evaluation, which is more reliable on examining fusion. Selection bias due to ambispective study was also a weakness. Moreover, intra- and inter-observer reliability for fusion assessment was not checked. There have been many articles on the topical of local vancomycin to reduce the risk of infection after orthopaedic surgery [3, 38, 39]. Moreover, vancomycin impregnated bone graft has been also widely used in spinal surgery [40–42]. Therefore, a prospective randomized study with an adequate patient number is needed to clarify the benefits of vancomycin impregnated autogenous bone graft and bone substitute on preventing DSSI following degenerative lumbar spine fusion surgery.

## Conclusions

Our study merely showed ABG with bone substitutes might be a local vancomycin delivery system to maintain high local concentration of vancomycin and to decrease DSSI incidences without detectable serum concentration and systemic adverse event, interfering posterolateral and interbody fusion or poor functional outcomes. Sterilized preparation, prophylactic antibiotics, environment and aseptic concepts for the staffs in the operating room are old fashion and still play important roles in preventing DSSI [43] and have to be emphasized besides application of local vancomycin.

## Data Availability

All data generated or analysed during this study are included in this published article.

## Abbreviations

DSSI: Deep surgical site infection
ABG: Autogenous bone graft
V: Vancomycin
NV: Non-vancomycin
VP: Vancomycin powder
TLIF: Transforaminal lumbar interbody fusion
