# Correction: Does local vancomycin powder impregnated with autogenous bone graft and bone substitute decrease the risk of deep surgical site infection in degenerative lumbar spine fusion surgery?—An ambispective study

**DOI:** 10.1186/s12891-023-06690-6

**Published:** 2023-07-22

**Authors:** Po‑Hsin Chou, Hsi‑Hsien Lin, Yu‑Cheng Yao, Ming‑Chau Chang, Chien‑Lin Liu, Shih‑Tien Wang

**Affiliations:** 1 School of Medicine, National Yang Ming Chiao Tung University, Taipei, Taiwan; 2 Department of Orthopedics and Traumatology, Taipei Veterans General Hospital, Taipei, Taiwan

**Correction to:** *BMC Musculoskelet Disord* **23, 853 (2022)**

10.1186/s12891-022-05802-y

Following publication of the original article [1], the authors reported typographic errors in the bottom half of Table 5 regarding the results for BSF definition. The correct table is given below.

**Table 5:** Results of bone fusion at latest follow-up between two groups

	Vancomycin (V)	No Vancomycin (NV)	P value
**Numbers of Patients**	110	86	
**Numbers of Discs with Cages Insertion**	132	100	
**Posterolateral Fusion Evaluated by Lenke Classification (of patients)**			0.563
A (Definite Solid)	40	31	
B (Possibly Solid)	29	24	
C (Probably Not Solid)	38	27	
D (Definitely Not Solid)	3	4	
**Interbody fusion evaluated by Brantigan, Steffee and Fraser definition (of cages)**			0.463
BSF-1 (radiographical pseudarthrosis)	**0**	**1**	
BSF-2 (radiographical locked pseudarthrosis)	11	10	
BSF-3 (radiographical fusion)	**121**	**89**	

+ 1 patient underwent cage removal surgery due to infective non-union and loosening during follow-up

The original article [1] has been corrected.
